# Comparative analysis of transcriptomic data shows the effects of multiple evolutionary selection processes on codon usage in *Marsupenaeus japonicus* and *Marsupenaeus pulchricaudatus*

**DOI:** 10.1186/s12864-021-08106-y

**Published:** 2021-10-30

**Authors:** Panpan Wang, Yong Mao, Yongquan Su, Jun Wang

**Affiliations:** 1 Jiangsu Key Laboratory of Marine Bioresources and Environment/ Jiangsu Key Laboratory of Marine Biotechnology, Jiangsu Ocean University, Lianyungang, 222005 China; 2 Co-Innovation Center of Jiangsu Marine Bio-Industry Technology, Jiangsu Ocean University, Lianyungang, 222005 China; 3 The Jiangsu Provincial Infrastructure for Conservation and Utilization of Agricultural Germplasm, Nanjing, 210014 China; 4 State Key Laboratory of Marine Environmental Science, College of Ocean and Earth Sciences, Xiamen University, Xiamen, 361102 Fujian China; 5 Fujian Key Laboratory of Genetics and Breeding of Marine Organisms, Xiamen University, Xiamen, 361102 China

**Keywords:** Codon usage pattern, *Marsupenaeus japonicus*, *Marsupenaeus pulchricaudatus*, Orthologous genes, Phylogenetics

## Abstract

**Background:**

Kuruma shrimp, a major commercial shrimp species in the world, has two cryptic or sibling species, *Marsupenaeus japonicus* and *Marsupenaeus pulchricaudatus*. Codon usage analysis would contribute to our understanding of the genetic and evolutionary characteristics of the two *Marsupenaeus* species. In this study, we analyzed codon usage and related indices using coding sequences (CDSs) from RNA-seq data.

**Results:**

Using CodonW 1.4.2 software, we performed the codon bias analysis of transcriptomes obtained from hepatopancreas tissues, which indicated weak codon bias. Almost all parameters had similar correlations for both species. The gene expression level (FPKM) was negatively correlated with A/T3s. We determined 12 and 14 optimal codons for *M. japonicus* and *M. pulchricaudatus*, respectively, and all optimal codons have a C/G-ending. The two *Marsupenaeus* species had different usage frequencies of codon pairs, which contributed to further analysis of transcriptional differences between them. Orthologous genes that underwent positive selection (*ω* > 1) had a higher correlation coefficient than that of experienced purifying selection (*ω* < 1). Parity Rule 2 (PR2) and effective number of codons (ENc) plot analysis showed that the codon usage patterns of both species were influenced by both mutations and selection. Moreover, the average observed ENc value was lower than the expected value for both species, suggesting that factors other than GC may play roles in these phenomena. The results of multispecies clustering based on codon preference were consistent with traditional classification.

**Conclusions:**

This study provides a relatively comprehensive understanding of the correlations among codon usage bias, gene expression, and selection pressures of CDSs for *M. japonicus* and *M. pulchricaudatus*. The genetic evolution was driven by mutations and selection pressure. Moreover, the results point out new insights into the specificities and evolutionary characteristics of the two *Marsupenaeus* species.

**Supplementary Information:**

The online version contains supplementary material available at 10.1186/s12864-021-08106-y.

## Background

The codon is the basic information unit for translation of messenger RNA (mRNA), and 62 codons encode 20 different amino acids [1–3]. For different genes or genomes, the selection of synonymous codons is nonrandom, which is called codon usage bias (CUB) [4, 5]. Codon preference is specific to the organism and may be influenced by GC content, gene expression level, and gene length [6–8]. In addition, codon usage patterns may affect the biological functions of mRNA biosynthesis, translation elongation rate, protein folding, and other downstream expressions [7, 9–12]. It is now thought that CUB is mainly affected by selection and mutational pressure [13–17]. Vicario et al. inferred that selection has acted on codon usage in the genus *Drosophila*, at least often enough to leave a footprint of selection in modern genomes [18]. Correspondence analysis proved that both selection and mutation pressure affect the codon usage pattern in *Bungarus* species [19]. Translational selection shapes codon and amino acid usage in three Pancrustacean arthropods [20]. In general, the pattern of codon usage is similar among closely related species but differs significantly among distantly related organisms [3, 18, 21–23]. Based on relative synonymous codon usage (RSCU) values, 27 species were clustered into two primary groups, which was consistent with the evolutionary status of these species [24]. According to these mentioned studies, codon usage showed evolutionary conservation and could be used for taxonomic differentiation.

The majority of past researches has studied the codon preference of species with genome-wide information [25–27]. Recent rapid development of next-generation sequencing has provided large amounts of genomic and transcriptome data. Machado et al., detected and quantified strong selection on synonymous sites of *Drosophila melanogaster* by using deep genomic population sequencing [28]. Utilizing Ribo-seq and RNA-seq approaches, Chu et al., studied how codon usage bias could impact the translation patterns of *Arabidopsis thaliana* [29]. Guan et al., analyzed codon usage of *Hirudinaria manillensis* RNA-seq data and found that genetic evolution was driven by mutation pressure and selection [30]. Based on the transcriptional sequence, Yi et al., found that the expression-linked patterns of codon usage revealed that higher expression was associated with higher GC_3_ and lower effective number of codons (ENC) [24]. Additional studies of codon usage bias based on transcriptome data include *Bombyx mori* [31], *Taenia multiceps* [32], and *Megalobrama amblycephala* [33].

The kuruma shrimp (*Marsupenaeus japonicus*) includes two cryptic species, distributed allopatrically but co-occurring in the northern South China Sea [34]. Previous studies showed obvious genetic differentiation between both shrimp species [35, 36]. Transcriptome analyses for these *Marsupenaeus* species evidenced a large number of putative orthologs, and the divergence time between *M. japonicus* and *M. pulchricaudatus* was approximately 0.26–0.69 Mya according to the peak of synonymous rates [37]. In *Arachis duranensis* and *Arachis ipaënsis*, Song et al., found the complex correlation among gene expression, codon usage bias, and substitution rate orthologs [38]. Orthologous genes typically perform equivalent functions across different species, which are closely related to gene expression [39]. However, the relationship between differentially expressed genes and codon usage patterns is still unknown in *Marsupenaeus* species.

This study performed codon usage bias analysis based on transcriptomes from *M. japonicus* and *M. pulchricaudatus* using CodonW software. We systematically compared the codon usage patterns of the two *Marsupenaeus* species and evaluated the comprehensive effects of various factors, including GC content, gene expression levels and gene length. The results provide new insights into the genetic divergence and the phylogenetic relationships of these two *Marsupenaeus* species.

## Results

### Nucleotide composition and PR2-plot analysis

**Fig. 1:**
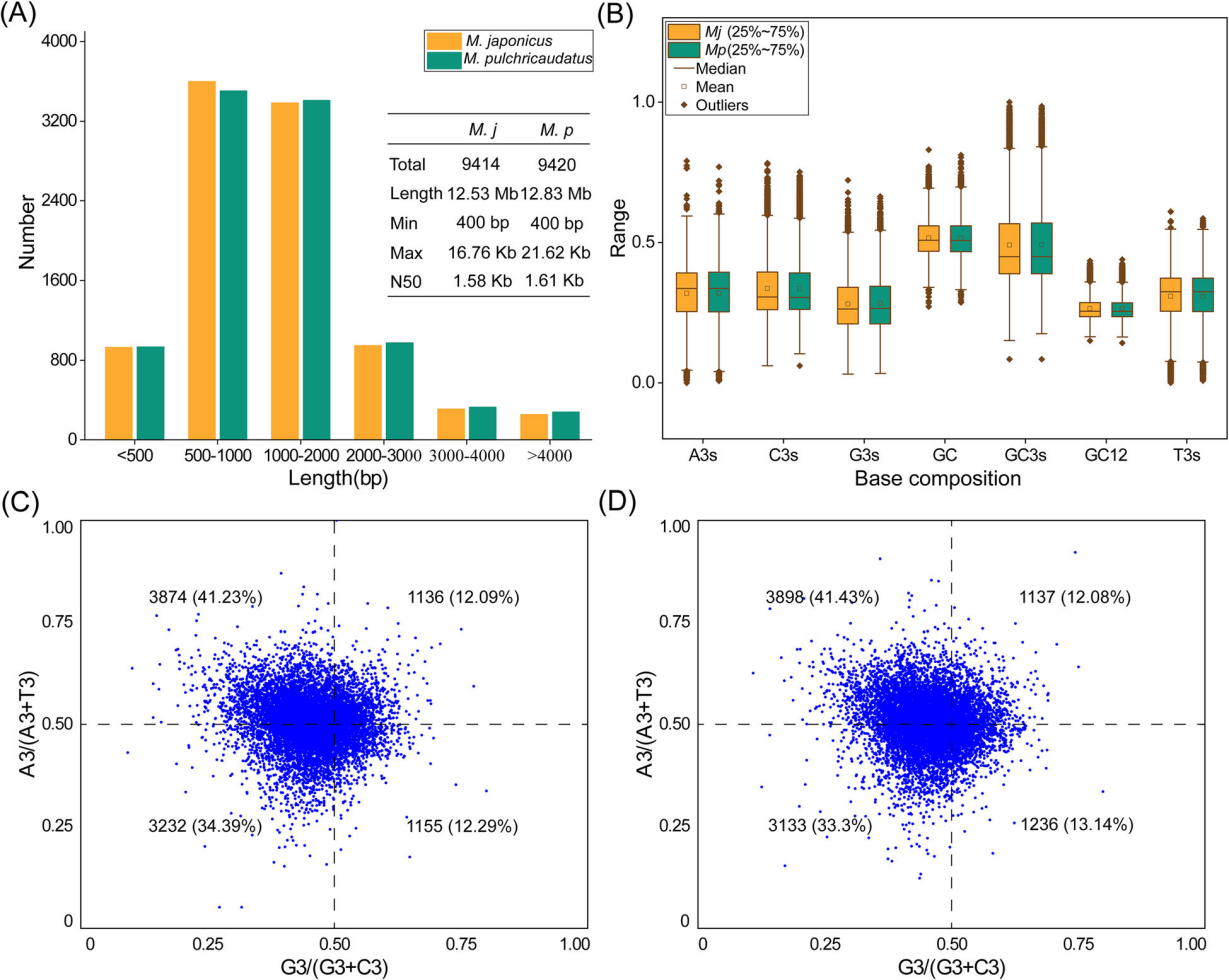
Length distribution of unigenes (a), nucleotide composition (b), and PR2 plot analysis for *M. japonicus* (c) and *M. pulchricaudatus* (d)

A total of 9414 and 9420 unigenes with lengths larger than 400 bp were screened from *M. japonicus* and *M. pulchricaudatus* libraries, respectively (Fig. 1a). The length distribution of the two groups was similar. In the *M. japonicus*, the mean contents of A and T nucleotides were 31.89% (SD = 10.45%) and 30.8% (SD = 9.04%), respectively, and the mean contents of C and G nucleotides were 33.63% (SD = 10.71%) and 28.03% (SD = 9.21%), respectively. In the *M. pulchricaudatus*, the average contents of A and T nucleotides were 31.86% (SD = 10.55%) and 30.8% (SD = 9.18%) respectively, and the average contents of C and G nucleotides were 33.56% (SD = 10.73%) and 28.27% (SD = 9.29%), respectively (Fig. 1b). The average contents of GC were 51.61 and 51.54% for *M. japonicus* and *M. pulchricaudatus*, respectively. The mean contents of GC3s were 49.1 and 49.17% for *M. japonicus* and *M. pulchricaudatus*, respectively, which were significantly higher than that of GC12. For *M. japonicus* and *M. pulchricaudatus*, the median of GC biases [G3/(G3 + C3)] were 0.4563 and 0.4582, and the median of AT biases [A3/(A3 + T3)] were 0.5047 and 0.5051, respectively (Fig. 1c, d). Parity Rule 2 (PR2) plot analysis showed that purines (A and G) were used more frequently than pyrimidines (C and T) in the two *Marsupenaeus* species. The unbalanced use of the third base suggested that mutation pressure and selection contribute to codon usage bias.

### Correlation analysis of codon usage parameters

**Fig. 2:**
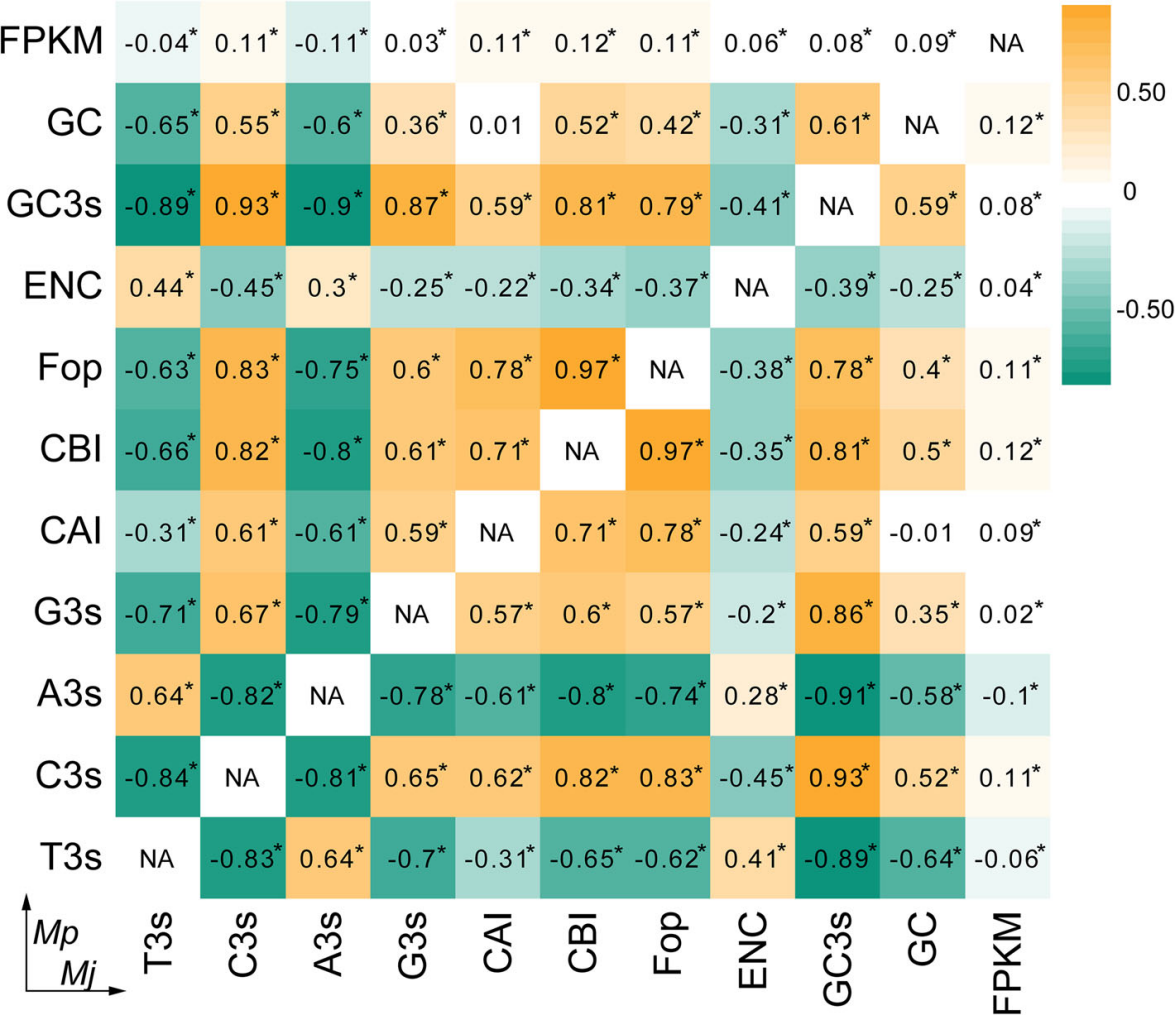
Correlation analysis of codon usage parameters. Significant difference at *p* < 0.05; ** significant difference at *p* < 0.01

All parameters had similar correlations between *M. japonicus* and *M. pulchricaudatus* (Fig. 2). The results indicated that FPKM was negatively correlated with T3s and A3s (*p* < 0.05) and positively correlated with other parameters (*p* < 0.05) in *M. japonicus* and *M. pulchricaudatus*. There was a significant (*p* < 0.05) positive correlation among T3s, A3s, and ENc values. These three values were negatively (*p* < 0.05) correlated with other parameters. Correlation analysis indicated that the third base content of synonymous codons significantly affects gene expression and codon usage bias. The significant correlation (*p* < 0.05) between GC3 and GC content indicated that the nucleotide contents play an important role in codon usage bias. The first and second base contents were often determined by selection and the third base content was affected by mutation pressure [40, 41].

The average ENc values were 52.1 and 52.22 for *M. japonicus* and *M. pulchricaudatus*, respectively. The number of genes with ENc values equal to 61was 268 (2.85%) and 249 (2.64%) for *M. japonicus* and *M. pulchricaudatus*, which indicates that all synonymous codons have the same probability. The number of genes with ENc values less than 35 was 187 (1.99%) for *M. japonicus* and 133 (1.41%) for *M. pulchricaudatus*, while the minimum values were 23.6 and 27.46, for these species, respectively. The S1|c21076_g1 unigene sequence had the lowest ENc, with 23.6 for *M. japonicus*. The gene was annotated as nesprins-1 (nuclear envelope spectrin repeat 1), a new member of the nuclear membrane protein family. The S2|c17052_g1 sequence had the lowest ENc with 27.46 for *M. pulchricaudatus*, which was annotated as the *vrille* (*vri*) gene.

A value of 35 was the standard for codon bias [42, 43]. To explore the effect of GC3s on codon usage bias, we performed ENc plot analysis. In Fig. S1A and S1B, most genes were aggregated close to the expected curve, which showed that codon usage bias was mainly affected by mutation pressure. We found lower ENc values in *M. japonicus* than in *M. pulchricaudatus*. Meanwhile, we estimated the difference between the expected and the observed ENc values and calculated the (ENcexp - ENcobs)/ENcexp (Fig. S1c, d). The frequency distribution of unigenes with values within 0–0.1 was highest, which showed that most ENc values from GC3s were larger than the observed ENc values. For *M. japonicus*, the average observed and expected ENc values were 52.1 and 56.67, respectively, and for *M. pulchricaudatus*, these values were 52.2 and 56.59, respectively. Moreover, there was a significant positive correlation between GC3s and CAI values (Fig. S1e, f).

### Gene ontology (GO) annotation based on GC3s

To further understand the influence of GC3s on gene function, we performed GO annotation for the CDSs with low, mid, and high GC3, including 1000, 1001, and 1005 genes in *M. japonicus* and 1005, 1001, and 1002 genes in *M. pulchricaudatus*. The gene ontology terms presented similar functional categories for both shrimp species (Fig. S2). The biological process categories, including 13 subtypes and most corresponding genes, were involved in cellular processes, metabolic processes, single-organism processes, and biological regulation. Thirteen subtypes were annotated with cellular component, and the highest gene number was observed in the “cell part” and “cell” categories. In the molecular function category, the “binding” was the highest category.

### Correlation analysis between codon usage parameters and the substitution rate

A total of 5036 pairs of single-copy orthologous genes were previously identified between the *M. japonicus* and *M. pulchricaudatus* libraries [37]. Among these orthologs, the *Ka/Ks* values of 2491 pairs were calculated, showing mean values equal to 0.002, 0.019, and 0.175 for *Ka*, *Ks,* and *Ka/Ks* (ω), respectively. There were 49 pairs of orthologous genes with a ω value greater than 1 (positive selection) and 2225 pairs with a ω value less than 1 (purifying selection).

**Fig. 3:**
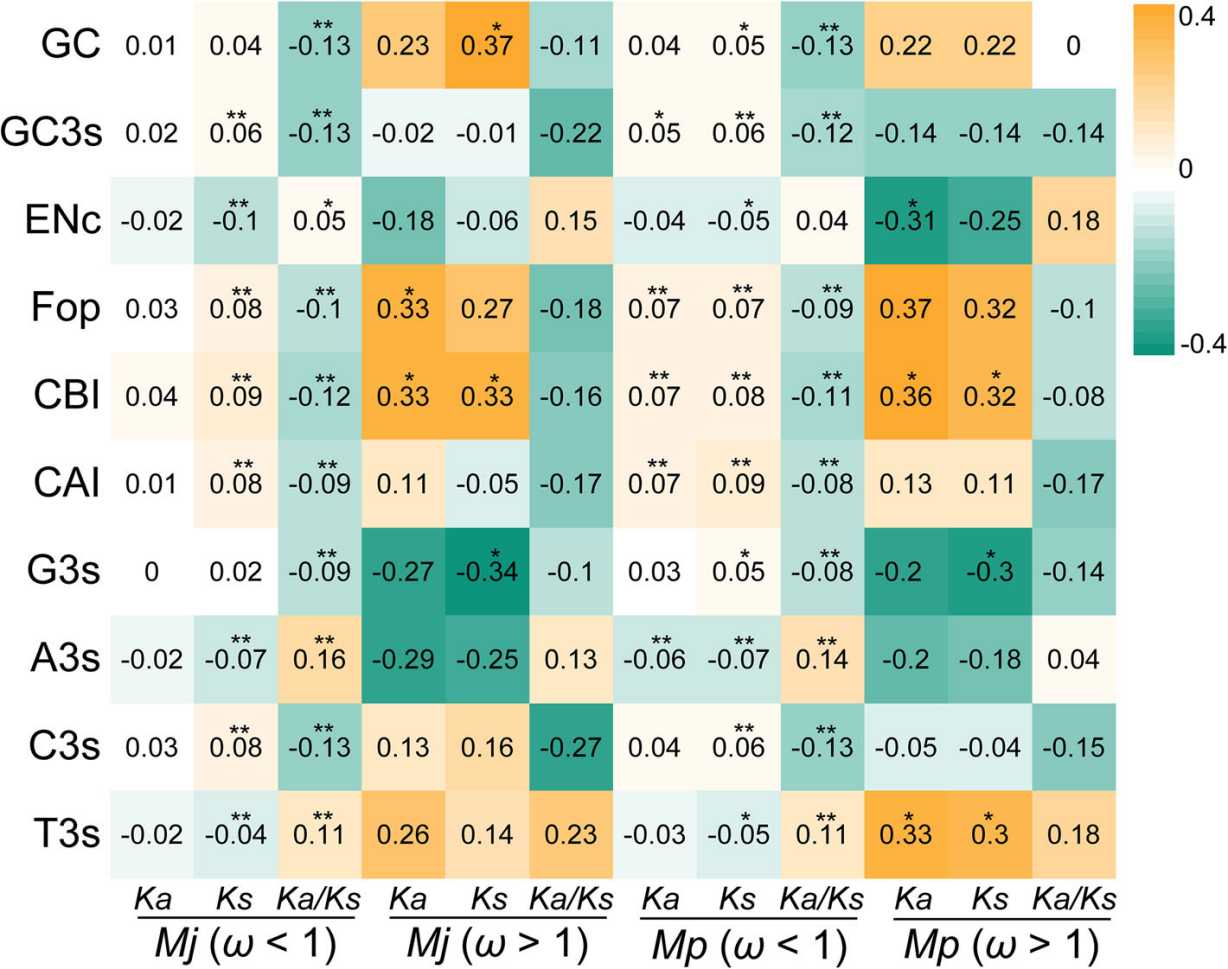
Correlation analysis between Ka and Ks values and codon preference. *Significant difference at *p* < 0.05; ** significant difference at *p* < 0.01

Overall, orthologous genes that underwent positive selection (ω > 1) had a higher correlation coefficient than those that experienced purifying selection (ω < 1), which could be because more genes with ω < 1 lead to large differences. Almost all parameters had different significance levels with *Ka*, *Ks,* or *Ka/Ks* (Fig. 3). In *M. japonicus*, the *Ka/Ks* of genes with ω less than 1 was positively correlated with ENc, A3s, and T3s (*p* < 0.01) but negatively correlated with other parameters (*p* < 0.01). There was no significant correlation between any parameters and the *Ka/Ks* of genes with ω greater than 1. However, GC content and CBI value were positively correlated with *Ks*, and G3s was negatively correlated with *Ks*. In addition, Fop and CBI values were positively correlated with *Ka*. In *M. pulchricaudatus*, the *Ka/Ks* of genes with ω less than 1 were positively correlated with A3s and T3s but negatively correlated with other parameters. Similar to *M. japonicus*, there was no significant correlation between all parameters and the *Ka/Ks* of genes with ω greater than 1. However, CBI and T3s values were positively correlated with *Ka* and *Ks*.

### Correspondence analysis (COA)

**Fig. 4:**
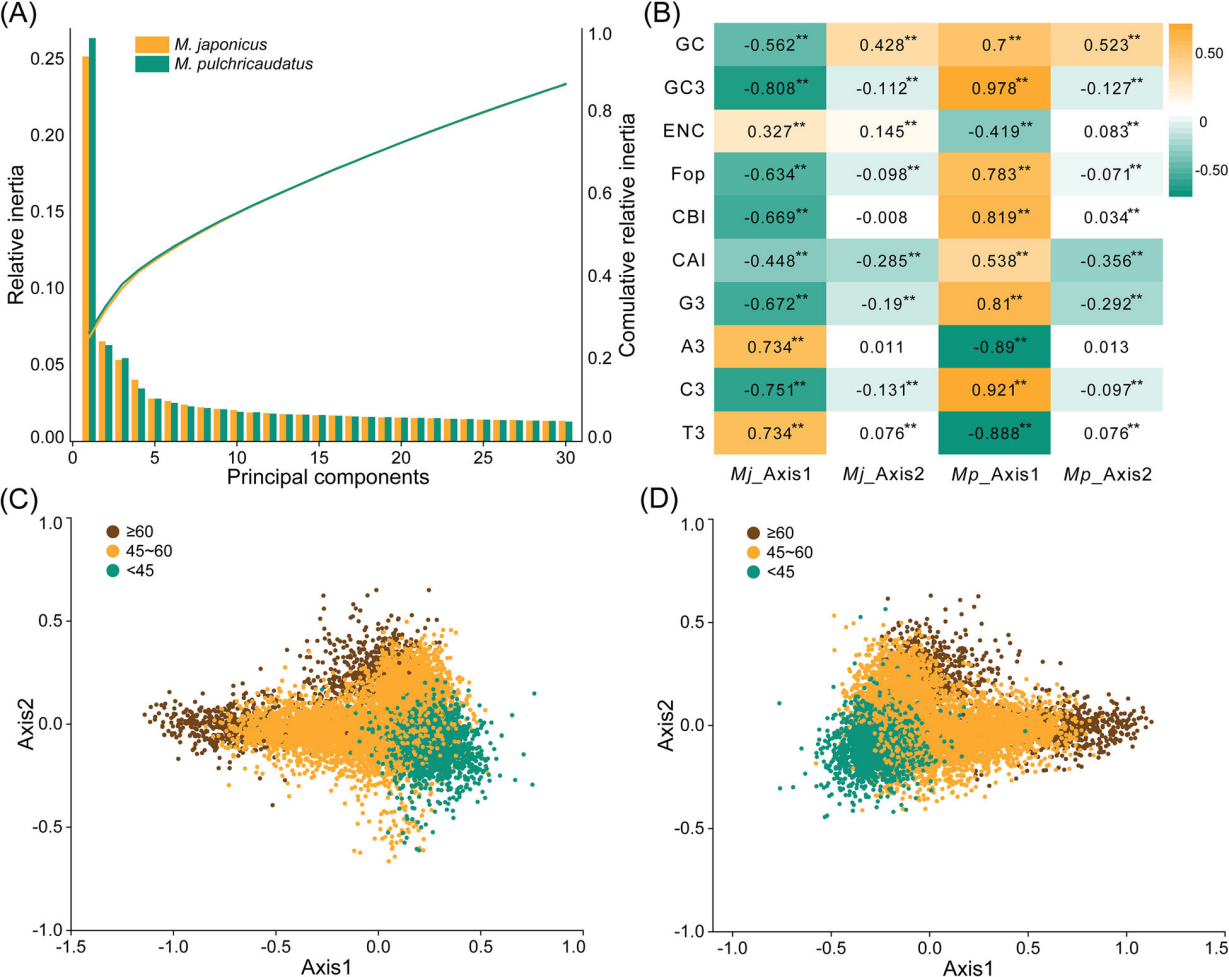
Correspondence analysis (a), correlation analysis (b) and GC content effect codon preference of *M. japonicus* (c) and *M. pulchricaudatus* (d). Different colors represent different GC contents, green represents GC% < 45%, yellow represents GC between 45and 60, and brown represents GC% > 60%

Based on the RSCU values, correspondence analysis was used to investigate the factors related to codon usage patterns and to reflect the variation trend in codon usage. The results indicated that the first five axes accounted for 43.8 and 44.3% of the amino-acid variation for *M. japonicus* and *M. pulchricaudatus*, respectively (Fig. 4a). In *M. japonicus*, Axis 1 and Axis 2 explained 25.16 and 6.54% of the variance, respectively. In *M. pulchricaudatus*, Axis 1 and Axis 2 explained 26.38 and 6.29% of the variance, respectively. In *M. japonicus*, the relationships were highly significantly positive between Axis 1 and A3, T3, and ENc (*p* < 0.01), and others were significantly negatively correlated (*p* < 0.01) (Fig. 4b). In *M. pulchricaudatus*, the relationships were highly significantly negative between Axis 1 and A3, T3, and ENc (*p* < 0.01) (Fig. 4b).

To identify the effect of GC content on codon bias, GC contents of genes were color-coded on the plot, which uses Axis 1 as the abscissa and Axis 2 as the ordinate (Fig. 4c for *M. japonicus* and Fig. 4d for *M. pulchricaudatus*). Overall, the distribution of GC content was the opposite along Axis 1. In *M. japonicus*, the larger the value of Axis 1, the smaller the GC content. The negative correlation (− 0.562 with *p*-value < 0.01) between Axis 1 and GC content is presented in the Fig. 4b. Instead, the larger the value of Axis 1, the larger the GC content of *M. pulchricaudatus*, and the positive correlation was 0.7 (*p* < 0.01).

### Determination of optimal codons

There were 32 codons with the RSCU values > 1 in *M. japonicas* and *M. pulchricaudatus*, which indicated that these codons were preferred by the two species (Table S1). Except for Trp and Met, the codons of Ala, Arg, Gly, Pro, Ser, and Thr had a higher bias. In addition, the codons with the RSCU value > 1 mainly ended with C and A. Based on ENc values, we obtained the RSCU datasets of high and low expression genes and calculated the △RSCU value (Table S2). We determined 12 and 14 optimal codons for *M. japonicus* and *M. pulchricaudatus*, respectively (Table 1). In *M. japonicus*, 9 optimal codons were C-ending, and 3 optimal codons were G-ending. In *M. pulchricaudatus* species, 9 optimal codons were C-ending, and 5 optimal codons were G-ending. Most optimal codons were the same in the two *Marsupenaeus* species, except ACC (Thr), CCG (Pro), GCG (Ala), and GGC (Gly).

**Table 1:** The optimal codons based on high and low levels of expression. AA: amino acids

		***M. japonicus***			***M. pulchricaudatus***		
**AA**	**Codon**	**RSCU-H**	**RSCU-L**	**△RSCU**	**RSCU-H**	**RSCU-L**	**△RSCU**
Val	GUC	1.557	0.995	0.563	1.454	0.984	0.470
Ser	UCG	1.005	0.682	0.324	1.202	0.717	0.486
Pro	CCC	1.552	0.906	0.646	1.764	0.920	0.844
	CCG	0.896	0.614	0.282	1.047	0.606	0.441
Thr	ACC	1.756	0.959	0.797	1.627	1.001	0.626
	ACG	1.170	0.698	0.472	1.356	0.720	0.636
Ala	GCG	0.836	0.564	0.271	1.002	0.537	0.464
Tyr	UAC	1.602	0.944	0.658	1.609	0.943	0.666
His	CAC	1.551	0.986	0.565	1.541	0.998	0.544
Asn	AAC	1.605	0.966	0.639	1.590	0.998	0.591
Asp	GAC	1.470	0.991	0.479	1.545	0.986	0.559
Glu	GAG	1.329	0.924	0.405	1.465	0.917	0.548
Cys	UGC	1.407	0.929	0.478	1.469	0.980	0.488
Arg	CGC	1.832	0.835	0.997	2.120	0.830	1.289
Gly	GGC	1.819	1.001	0.818	2.108	0.978	1.130

### Codon pairs in two *Marsupenaeus* species

A synonymous codon that encodes two amino acids is called a duplex codon or codon pairs and is more commonly used than a single codon. The two *Marsupenaeus* species had different use frequencies of codon pairs (Table 2), such as GlyAla (GGAGCU vs GGAGCA), GlnArg (CAGAGA vs CAAAGA), and GluAsn (GAGAAC vs GAAAAU). In *M. japonicus*, the high-frequency codon pair of ArgArg was AGAAGA, while the optimal codon of Arg was CGC (Fig. S3). The high-frequency codon pair of AspAsp was GAUGAU, while the optimal codon of Asp was GAC. The high-frequency codon pair of GluGlu was GAAGAA, while the optimal codon of Glu was GAG. There were other inconsistencies, including GlyGly (GGAGGA) and Gly (GGC), HisHis (CAUCAU) and His (CAC), ProPro (CCACCA) and Pro (CCC), SerSer (AGCAGC) and Ser (UCG), ThrThr (ACAACA) and Thr (ACC/ACG), and ValVal (GUGGUG) and Val (GUC) (Fig. S3). In *M. pulchricaudatus*, the high-frequency codon pair of HisHis was CACCAC, which differentiates it from that of *M. japonicus*. The high-frequency codon pair of ProPro was CCACCA, while the optimal codons of Pro were CCC and CCG. The high-frequency codon pair of AlaAla was GCAGCA, while the optimal codon of Ala was GCG (Fig. S4). Codon pair utilization biases play an important role in protein synthesis by interacting with tRNA isoacceptors [44]. Codon pair analysis enables us to obtain a clear picture of the codon usage bias during transcription and translation.

**Table 2:** The different duplex codons of two *Marsupenaeus* species

Codons	***M. japonicus***	***M. pulchricaudatus***	Codons	***M. japonicus***	***M. pulchricaudatus***
Arg_Pro	AGGCCA	AGACCA	Phe_Thr	TTCACA	TTCACC
Asn_Ile	AACATT	AACATC	Pro_His	CCTCAT	CCTCAC
Asn_Leu	AACCTC	AACCTG	Pro_Lys	CCCAAG	CCAAAG
Asp_Pro	GATCCA	GACCCA	Pro_Ser	CCTTCA	CCATCA
Asp_Val	GATGTG	GATGTT	Pro_Val	CCAGTG	CCTGTG
Cys_Met	TGTATG	TGCATG	Ser_Gln	TCACAG	AGCCAG
Gln_Arg	CAGAGA	CAAAGA	Ser_Met	TCCATG	TCAATG
Gln_Ile	CAGATC	CAGATT	Thr_Leu	ACCCTC	ACTTTG
Glu_Asn	GAGAAC	GAAAAT	Trp_His	TGGCAC	TGGCAT
Gly_Ala	GGAGCT	GGAGCA	Trp_Tyr	TGGTAC	TGGTAT
Gly_Val	GGAGTG	GGTGTT	Tyr_Ala	TATGCT	TATGCA
His_His	CATCAT	CACCAC	Tyr_Pro	TACCCA	TATCCA
Leu_Leu	CTGCTG	CTCCTC	Val_Cys	GTGTGC	GTGTGT
Leu_Met	CTGATG	TTGATG	Val_Gly	GTGGGC	GTTGGA
Lys_Glu	AAAGAA	AAGGAA	Val_Ser	GTGTCA	GTCAGC
Phe_Ala	TTTGCA	TTTGCT			

### Multispecies clustering analysis

**Fig. 5:**
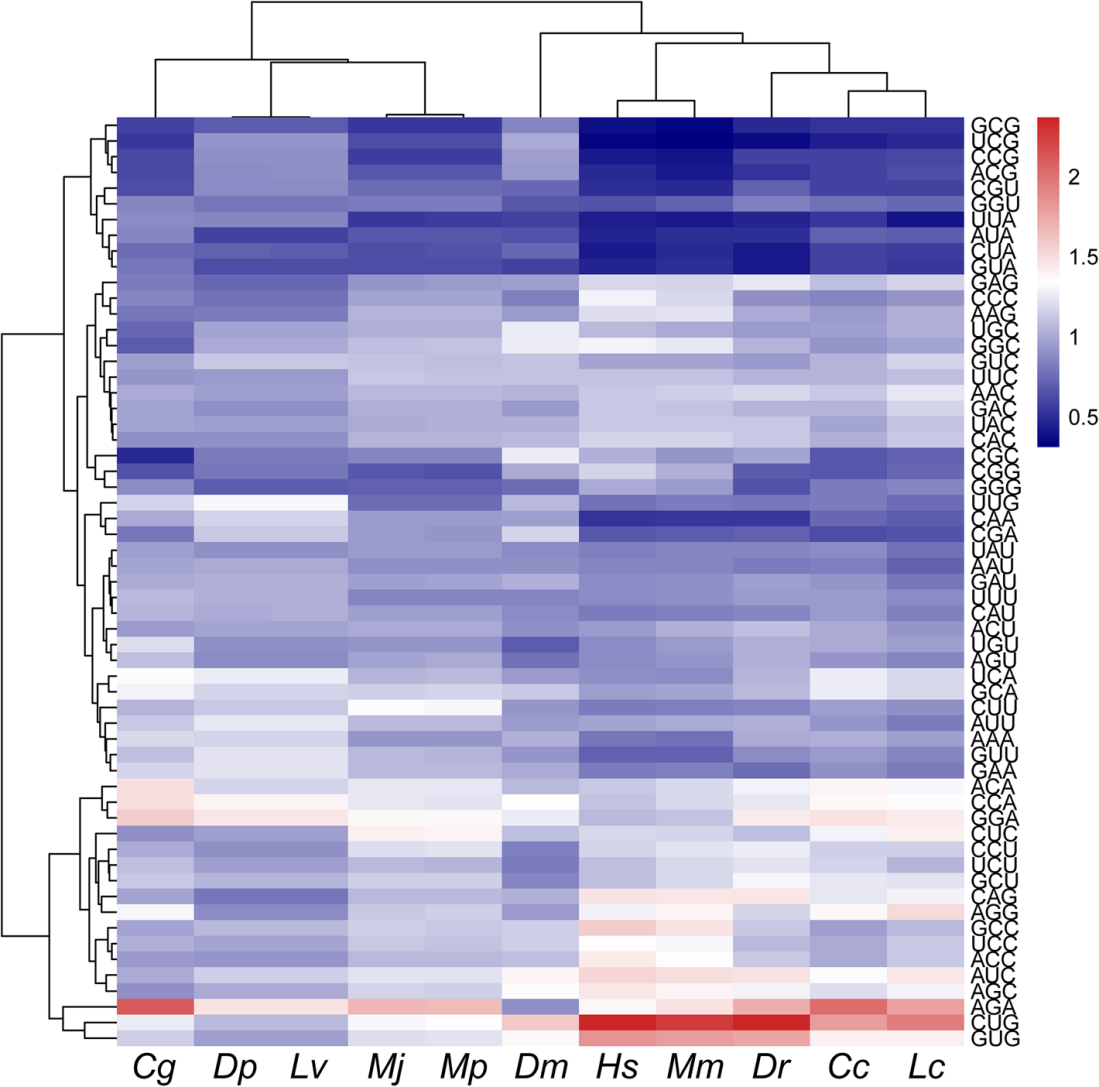
Clustering analysis based on RSCU values. Cg = *Crassostrea gigas*, Dp = *Daphnia pulex*, Lv = *Litopenaeus vannamei*, Mj = *Marsupenaeus japonicus*, Mp = *Marsupenaeus pulchricaudatus*, Dm = *Drosophila melanogaster*, Hs = *Homo sapiens*, Mu = *Mus musculus*, Dr. = *Danio rerio*, Cc = *Cyprinus carpio*, Lc = *Larimichthys crocea*

**Fig. 6:**
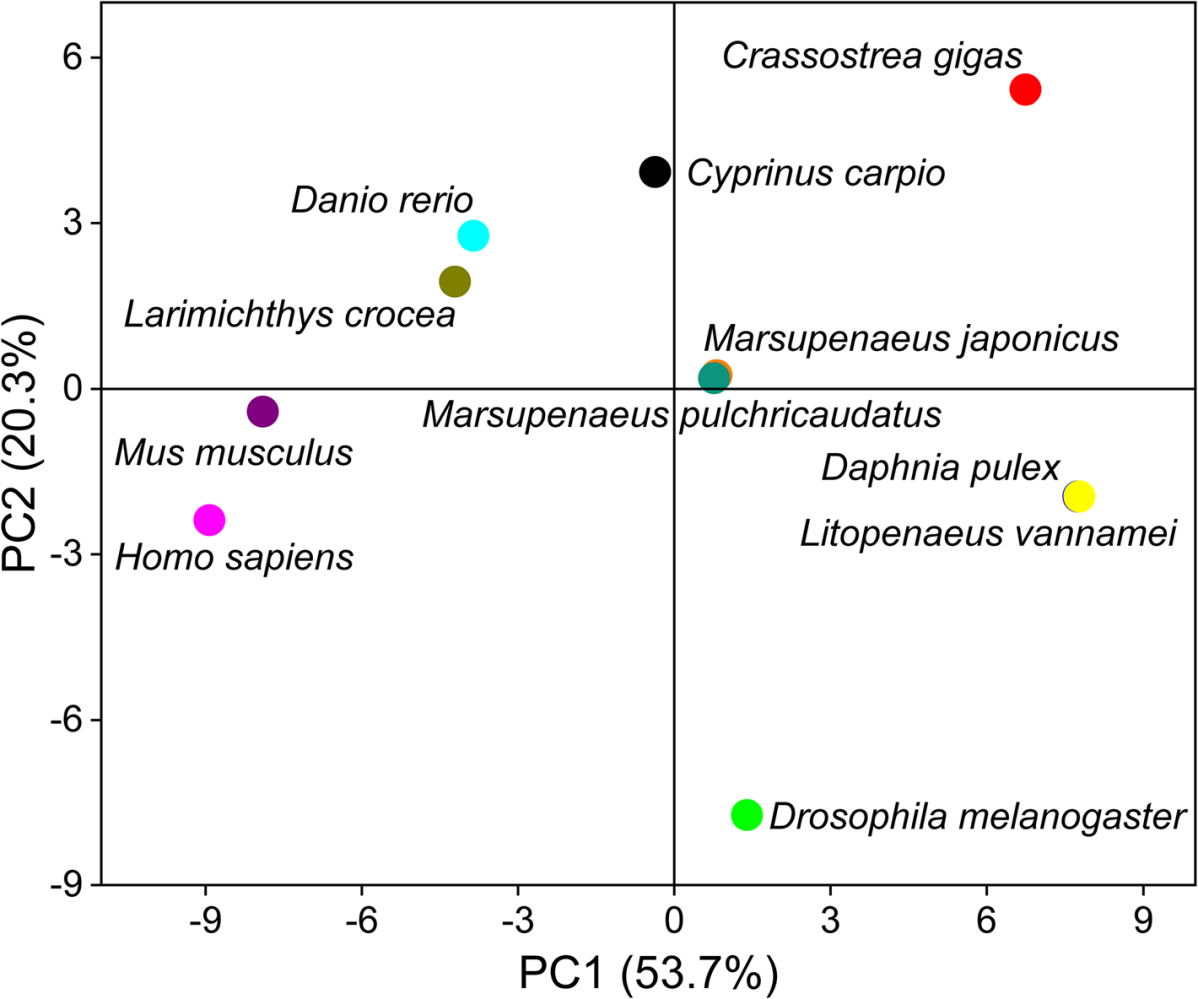
Principal component analysis (PCA) of RSCU values

Based on the RSCU values of 59 codons (except Met, Trp, Taa, Tag, and Tga), the heat map (Fig. 5) showed that two *Marsupenaeus* species were clustered with *Daphnia pulex* and *Litopenaeus vannamei* and then *Crassostrea gigas*. The *Larimichthys crocea*, *Cyprinus carpio,* and *Danio rerio* were classified into the same cluster. *Homo sapiens* and *Mus musculus* were clustered into one group. Interestingly, *Drosophila melanogaster* and mammals were grouped at first, and then Arthropoda and *Crassostrea gigas* joined in them. This may be mainly because *D. melanogaster* has a stronger codon preference than other arthropods. Similar to the clustering results, the PCA showed that the two *Marsupenaeus* species overlapped almost completely, and the relationship between *C. gigas* and arthropods was not as strong as indicated by the results of heat map clustering (Fig. 6). These clustering results were consistent with traditional species classification.

## Discussion

Given the significant biological effects of different codon patterns, identifying these patterns in a given gene or genome is important to understand the molecular mechanisms of gene expression and to uncover the effects of long-term evolution on the genome [15, 45, 46]. Moreover, identifying these patterns is helpful for the phylogenetic analysis of species and to improve the expression of a target gene by optimizing codons [23, 47–49].

In this study, we analyzed the codon preferences of transcripts of two *Marsupenaeus* species, which were consistent overall. There was no significant difference in the content of AT and GC of the third base. The first and second base contents of a codon are usually affected by selection, while the third base content is affected by mutation pressure [40, 41]. The gene expression level (FPKM) was significantly negatively correlated with A/T3s. This result indicated that the third codon base significantly affects codon preference and gene expression level. Many studies have shown that codon usage bias correlates with gene expression levels, and codon usage patterns of highly expressed genes affect proteome-wide translation efficiency [12, 50, 51]. Whittle et al. found that translational selection shapes codon and amino acid usage in three Pancrustacean arthropods [20]. In *Parasteatode tepidariorum*, highly expressed genes favored amino acids with low or intermediate size/complexity (S/C) scores (glycine and alanine) and disfavored those with large S/C scores (such as cysteine) [50]. Further studies must consider correlation analysis between codon usage, amino acid frequency and expression levels.

The mean effective codon numbers (ENc) of the two cryptic species were 52.1 and 52.2, respectively, indicating the weak codon preference of both species. The S1|c21076_g1 sequence had the lowest ENc, with 23.6 for *M. japonicus*, and was annotated as *nesprins-1*, which is involved in the formation of the gamete cytoskeleton at different developmental stages [52]. Our previous study showed that there was significant variation in spermatheca traits, including the ratio of spermatheca length and width to body length. It remains to be further verified whether the dynamic expression level of this gene is different in the same developmental period of both species. The S2|c17052_g1 sequence, with the lowest ENc for *M. pulchricaudatus*, was annotated as the *vrille* (*vri*) gene, which encodes a core transcriptional repressor required for circadian behavior in *Drosophila* [53]. The two *Marsupenaeus* species have distinct geographical features with significant environmental differences, including temperature, sunlight and ocean currents. Long-term selection effects of different environments may affect the biorhythm, which still needs to be further tested. The FPKM of the S1|c21076_g1 and the S2|c17052_g1 sequences indicated low and high expression levels, respectively. Genes using the codons that are recognized by more abundant tRNA molecules may be translated more efficiently and with fewer mistakes than genes that use less frequent codons [54, 55]. Nelson et al. found that the high frequency of AGA/AGG codons present in the HCcAg and _HU_IFNa2 genes could be one of the factors limiting its expression in *Escherichia coli* [47]. In future studies, we will consider measuring the tRNA gene copy numbers and performing the correlation analysis with gene expression levels.

The codon preference of different species is generally influenced by mutation and selection pressure [56, 57]. The PR2 analysis showed that the usage frequencies of the four bases were not equal in the two *Marsupenaeus* species, suggesting that mutation pressure and selection contribute to codon usage patterns [58]. The ENc-GC3 plot reflects underlying factors governing CUB, which is based on the assumption that only GC content determines variations [59]. When the codons are affected only by GC compositional constraints, the gene lies on or very close to the curve. In the present study, the average observed ENc value was lower than the expected value for both species, suggesting that factors other than GC may act. Hiroshi Akashi et al. conducted seminal studies using population genetic approaches to corroborate the major codon preference model in *Drosophila*, which showed that selection does indeed affect the silent sites of proteins [60]. Based on 75 orthologous gene pairs from *Drosophila*, McVean and Nielsen estimated parameters of both mutation and selection, and the results showed considerable variation in the strength of selection between different *Drosophila* species [61].

Overall, the correlation analysis between the *Ka*/*Ks* value and codon preference parameters of orthologous genes in the two cryptic species was consistent. Nielsen et al. used a more complex mutation model to simultaneously estimate mutation rates, dN/dS, and the results supported the major codon preference model, and the *notch* gene of *Drosophila melanogaster* showed evidence of selection on synonymous sites [62]. In the group with ω < 1, the *Ka/Ks* of variety I was significantly positively correlated with ENc. In the group with ω > 1, *Ka* and *Ks* were significantly positively correlated with T3s, and the Fop and GC contents of variety I were significantly positively correlated with *Ka* and *Ks*, respectively. In *Arachis duranensis* and *Arachis ipaënsis* orthologs, highly expressed genes were subjected to stronger selective pressure than genes with low expression levels based on the negative correlation between selection constrain and both gene expression [38]. The positively selected orthologous genes related to the immune process mainly comprised single Von Willebrand factor, type C (VWC) domain protein, legumain, ras-related C3 botulinum, caspase, protein kinases, profilin family protein [37]. These genes were mainly annotated with the GO terms biological process (innate immune response, response to abiotic or biotic stimulus). The main reason for selection of codon bias may be that the increased use of major codons leads to more efficient and more accurate translation. However, some genes have been found to be under selection in the opposite direction, and the exact relative contribution of selection remains unclear [63]. The results of the correspondence analysis showed that the codon preference parameters of the two cryptic species had an opposite correlation with axis 1, which has been considered the most important evaluation index, and here showed a highly significant correlation with C3 and GC3. The gene expression level was significantly positively correlated with GC content. GC content is likely to be determined mostly by genome-wide processes rather than by selective forces acting specifically on coding regions, being the most significant parameter explaining codon bias differences between different organisms [64]. The results from Camiolo et al., indicated that gene sequences with higher GC content showed a higher expression level and better codon preference [65]. More efficient transcription and translation by the use of optimal synonymous codons increases the fitness of the organism [66, 67].

In this study, RSCU and ENc values were combined to determine 12 optimal codons in variety I, among which 9 ended in C and 3 ended in G, and 14 optimal codons in variety II, among which 9 ended in C and 5 ended in G. These results showed that *Marsupenaeus* species are genetically more likely to end in C/G, which was similar to the codon usage characteristic of carp (*Cyprinus carpio*), zebrafish (*Danio rerio*), *Acanthopagrus schlegelii* and *Pagrus major* [68, 69]. This may be because the evolution of *M. japonicus* is mainly mutated from AT to CG. Based on RNA-seq data, Whittle et al. found that three Pancrustacean arthropods have different optimal codons in highly expressed genes, and the majority of optimal codons from *Parhyale hawaiensis* were GC3 codons [20]. In *Parasteatoda tepidariorum*, highly expressed genes exhibited preferential usage of T3 codons, suggestive of selection [50]. Al-Saif et al., showed that reducing the proportion of UU or UA could enhance the resistance to mRNA attenuation, thus increasing protein expression [70]. In recent decades, the roles of codon usage bias in fine-tuning transcription, post-transcriptional processing, mRNA stability, translation initiation, elongation, and peptide folding have been revealed. The expression of functional proteins in heterologous hosts is a cornerstone of modern biotechnology, and the existence of slightly different codes in different organisms is a very significant barrier to heterologous expression [49]. The peptide LBDv (lipopolysaccharide binding domain) was synthesized based on the modified sequence of LBD (named LBD2) from FcALF2 and exhibited an apparently enhanced antimicrobial activity [71]. There were 31 different double codon pairs between the two *Marsupenaeus* species, and the optimization of the codon pair could improve the efficiency of protein translation compared with the single optimal codon [72–74]. The genetic distance of species is closely related to the codon preference difference, which can be used for species classification [33]. Based on the RSCU values of mitochondrial genomes among shrimp, the multidimensional scaling (MDS) plot showed that, for the most part, members of each infraorder clustered together and were largely distinct from the samples from the other infraorders [75]. The results of multispecies heat map analysis and clustering based on RSCU values are consistent with traditional species classification, which supported our previous results based on genotyping-by sequencing (GBS) and single copy nuclear genes (SCNGs) [35]. The results indicated that the size of interspecies codon preference differences can reflect the proximity of species, which is also verified in other species [21, 74, 76, 77].

## Conclusions

In conclusion, we systematically compared the codon usage patterns of two *Marsupenaeus* species and evaluated the comprehensive effects of various factors. The codon usage patterns of both species were affected by mutations and selection. This study provides a relatively comprehensive understanding of the correlations among codon usage bias, gene expression, and selection pressure of CDS from *M. japonicus* and *M. pulchricaudatus*. Moreover, the results point out new insights into the specificities and evolutionary characteristics of these two cryptic species. However, the effect of codon usage bias on gene expression and the biological implications of different optimal codons in both species need further exploration.

## Methods

### Data collection and filtering

cDNA libraries were constructed from hepatopancreas of ten healthy *M. japonicus* (weight: 12.67 ± 3.22 g) and ten healthy *M. pulchricaudatus* (weight: 11.36 ± 4.2 g) from Huilai (Guangdong, China), and then sequenced for transcriptome assembly and functional annotation, as previously reported [37]. Raw Illumina sequences are accessible from NCBI Sequence Read Archive (SRA) (https://trace.ncbi.nlm.nih.gov/Traces/sra/) under accession SRR7786082 (*Marsupenaeus pulchricaudatus*) and SRR7786083 (*Marsupenaeus japonicus*). A total of 14,126 and 13,695 unigenes with CDS regions were identified from the *M. japonicus* and *M. pulchricaudatus* libraries, respectively. Orthologous groups were screened using OrthoMCL with default settings [78]. Gene expression levels as fragments per kilobase million [79] were estimated by RSEM software [80]. Coding sequences of the other nine species (Table S3) were downloaded from NCBI (https://www.ncbi.nlm.nih.gov/). All CDSs were filtered using the OmicShare online platform (http://www.omicshare.com/tools), and those sequences with lengths less than 400 bp or unknown bases were eliminated.

### Codon usage indices analysis

The GC1, GC2, and GC3 contents were calculated using Perl GitHub, and GC12 was the average value of GC1 and GC2. Using the CodonW 1.4.2 software (http://codonw.sourceforge.net), we performed codon bias analysis. The calculation indices included GC content, nucleotide composition at the 3rd codon position (A3s, T3s, G3s, and C3s), effective number of codons (ENc), the codon adaptation index (CAI), codon bias index (CBI), frequency of optional codon (Fop), and relative synonymous codon usage (RSCU), and so on. The parity rule 2 (PR2) plot analysis was based on the third codon position, using A3/(A3 + T3) as the ordinate and G3/(G3 + C3) as the abscissa. The PR2 plot can be used to estimate the impact of selection and mutation pressure on codon usage bias [81].

### ENc-plot and GO annotation

The effective number of codons (ENc), with a value between 20 and 61, is a key parameter to interpret codon bias. The value 20 indicates that only one synonymous codon is chosen, 61 represents no usage bias, and all synonymous codons have the same probability. The lower the value for a coding sequence, the stronger the codon usage bias [42, 82]. In general, a gene possesses strong codon usage bias when the ENc value is lower than 35 [43, 83]. The ENc plot was drawn by Origin 2020 (OriginLab Corporation, USA), which uses the ENc value as the ordinate and GC3s as the abscissa. The expected ENc values were calculated based on the equation: Enc (exp) = 2 + GC3s + 29/[GC3s ^2^ + (1- GC3s)^2^] [84]. The codon adaptation index (CAI) is an important index for estimating synonymous codon usage bias and gene expression levels, and a higher CAI value signifies the stronger codon usage bias [85–87]. Gene ontology annotation was performed using Blast2GO v2.5 (*E*-value <1e^− 6^) [88]. GO classifications were compared among different groups of GC3s (High, Mid, and Low) using the OmicShare online platform.

### Correlation analysis

The codon usage patterns were often shaped by many factors, such as GC content, expression level, tRNA abundance, protein structure, and hydrophilicity [89, 90]. We performed a correlation analysis between codon bias parameters and expression level (FPKM). Using the PAML toolkit [91], we calculated the nonsynonymous substitution ratio (*Ka*) and synonymous substitution ratio (*Ks*). The *Ka/Ks* (ω) can be used to determine whether there is selective pressure on protein-encoding genes [92, 93]. Values of ω > 1 suggest that the gene evolved under positive selection, whereas ω close to zero indicates that the gene is under heavy selection pressure [92, 94].

### Correspondence analysis (COA)

To further investigate the factors related to the codon usage pattern, correspondence analysis was conducted by CodonW based on the RSCU values. The COA was used to compare the usage patterns of 59 codons (except Met, Trp, Taa, Tag, and Tga) and reflect the variation trend in codon usage. COA creates a series of orthogonal axes, which were used to estimate the main source of variation. Using SPSS v22 (https://www.ibm.com/support/pages/spss-statistics-220-available-download), the relative coefficient between ten codon bias parameters and Axis1 and Axis2 was calculated.

### Relative synonymous codon usage and optimal codons

According to Sharp et al. [95], the relative synonymous codon usage (RSCU) is an index to measure the codon usage preference. The higher the RSCU value, the stronger the preference. Based on the calculated ENc values, 10% of the genes with extremely high and low ENc values were regarded as the high and low RSCU datasets [96]. The optimal codons were confirmed based on the △RSCU value and chi-square test [66, 83, 97].

### Clustering and principal component analysis

The protein-coding sequences of nine species (Table S3) were downloaded from the ensemble database (http://asia.ensembl.org/index.html) and NCBI (https://www.ncbi.nlm.nih.gov/), and codon usage preference was analyzed using CodonW. The heatmap was generated based on RSCU values using the OmicShare online platform. Based on the RSCU of 59 codons, principal component analysis (PCA) was performed using Origin 2020 (OriginLab Corporation, Northampton, MA, USA).

## Supplementary Information

**Additional file 1: Fig. S1.** ENc plot, ENc frequency and GC3s-CAI for *M. japonicus* (a, c, e) and *M. pulchricaudatus* (b, d, f).

**Additional file 2: Fig. S2.** Gene ontology (GO) annotation.

**Additional file 3: Fig. S3.** The codon pairs of *M. japonicus.*

**Additional file 4: Fig. S4.** The codon pairs of *M. pulchricaudatus*.

**Additional file 5: Table S1.** The relative synonymous codon usage (RSCU) of synonymous codons. **Table S2.** The RSCU datasets of high and low expression genes. **Table S3.** Genome information of nine species.

## Data Availability

All the necessary information needed to support the results of this paper are included within the article. All of the RNA sequencing data used in the study are available through the NCBI SRA database (https://trace.ncbi.nlm.nih.gov/Traces/sra/) under the accession numbers SRR7786082 (*Marsupenaeus pulchricaudatus*) and SRR7786083 (*Marsupenaeus japonicus*). The protein-coding sequences of other species (Table S3) were downloaded from the ensemble database (http://asia.ensembl.org/index.html) and NCBI (https://www.ncbi.nlm.nih.gov/) with accession numbers: PRJNA629593; PRJEB14656; PRJNA508983; PRJNA164; PRJNA168; PRJNA169; PRJNA13922; PRJNA352247; PRJNA354443.

## Abbreviations

NCBI: National Center for Biotechnology Information
GO: Gene Ontology
CDS: the coding sequences
SRA: Sequence Read Archive
ENc: the effective number of codons
CAI: the codon adaptation index
CBI: codon bias index
Fop: frequency of optional codon
RSCU: relative synonymous codon usage
COA: correspondence analysis
PR2: parity rule 2
FPKM: Fragments Per Kilobase per Million
PCA: principal component analysis
Ka: non-synonymous substitution ratio
Ks: synonymous substitution ratio
