# Supplementary figures and images for: Comparative analysis of transcriptomic data shows the effects of multiple evolutionary selection processes on codon usage in Marsupenaeus japonicus and Marsupenaeus pulchricaudatus
Source: BMC Genomics. 2021 Oct 30;22:781. doi: 10.1186/s12864-021-08106-y (PMC8557549; doi:10.1186/s12864-021-08106-y)

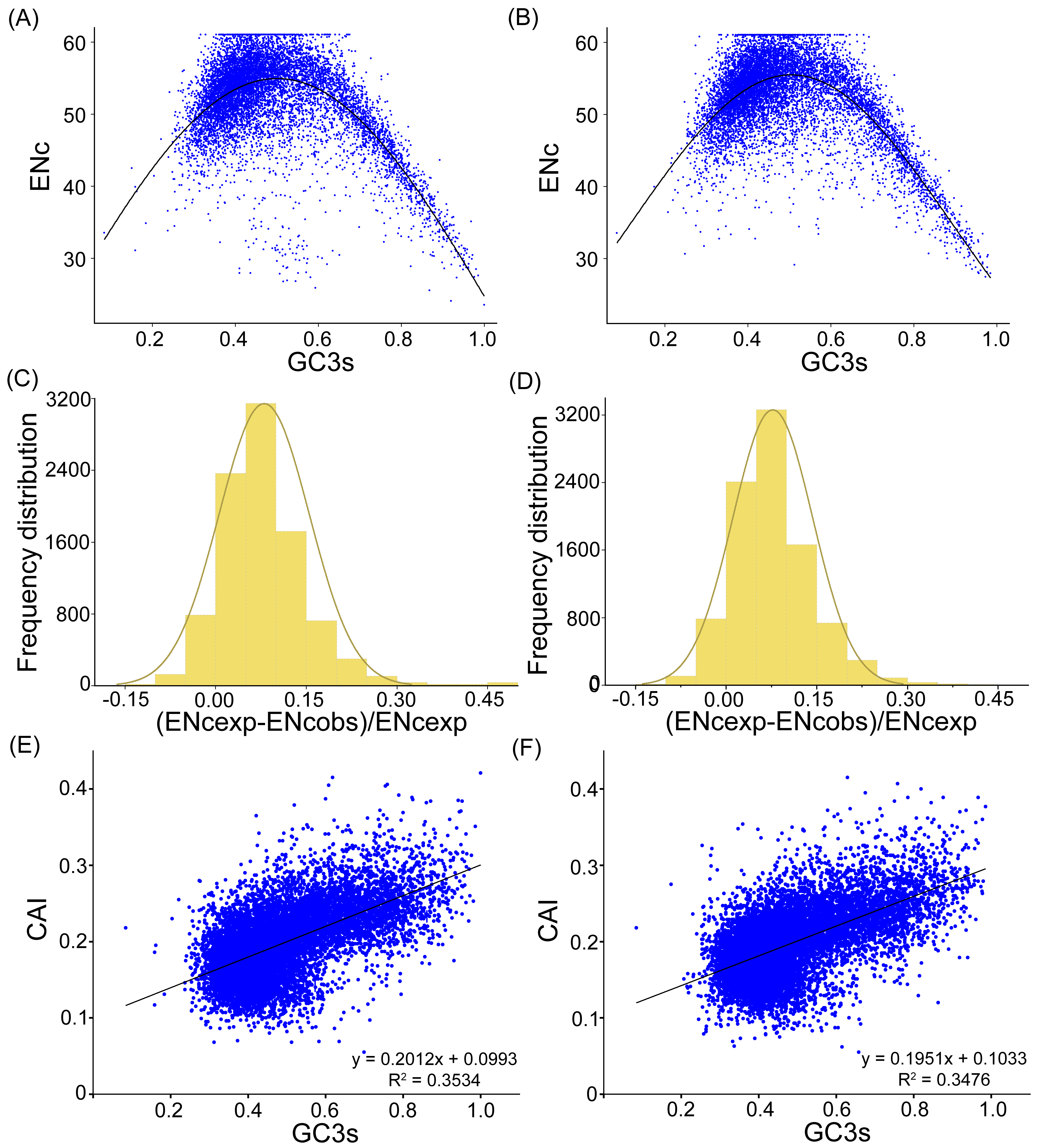

Supplement: Supplementary file 1 — Additional file 1: Fig. S1. ENc plot, ENc frequency and GC3s-CAI for M. japonicus (a, c, e) and M. pulchricaudatus (b, d, f). [file 12864_2021_8106_MOESM1_ESM.tif]

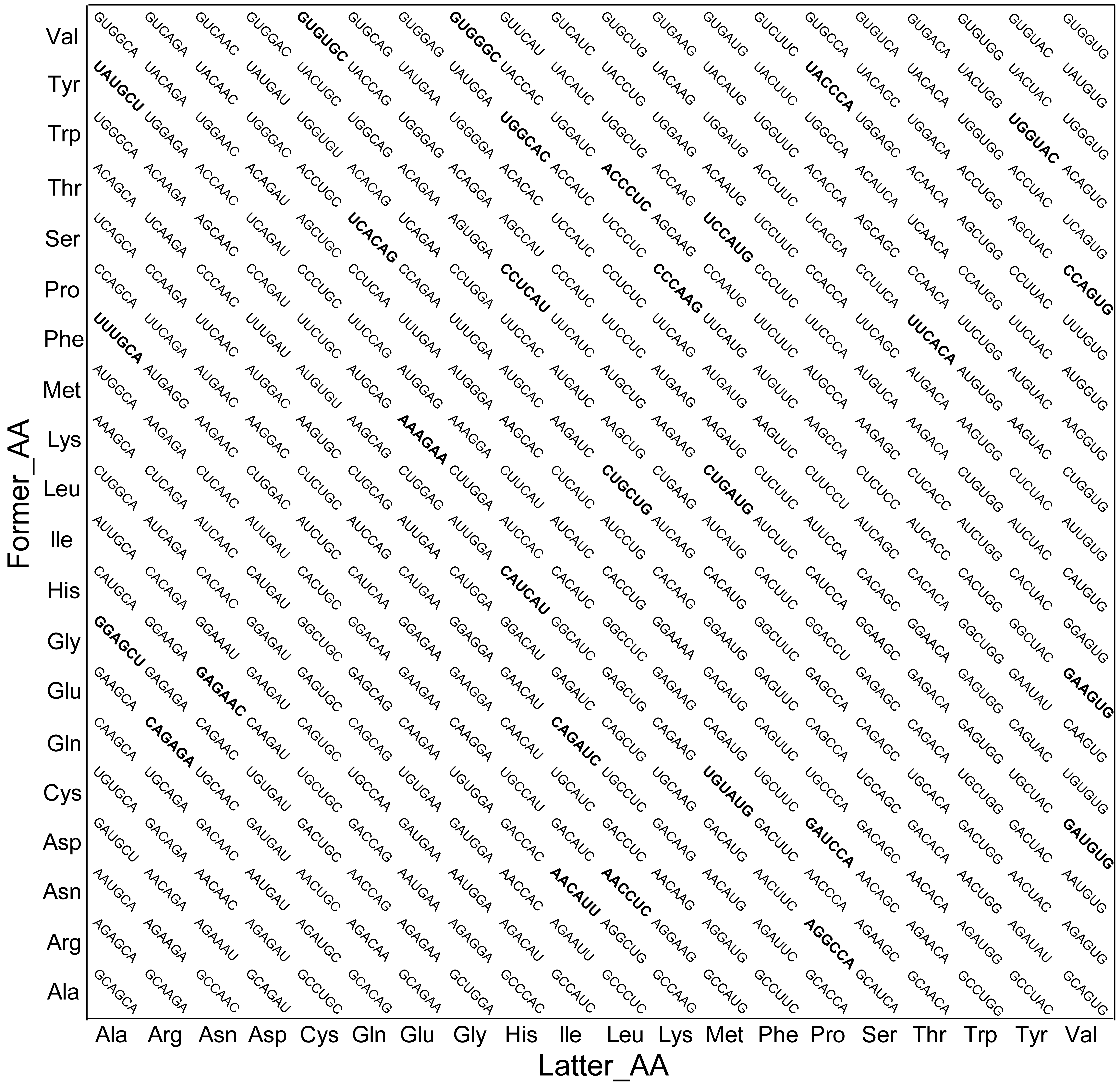

Supplement: Supplementary file 3 — Additional file 3: Fig. S3. The codon pairs of M. japonicus. [file 12864_2021_8106_MOESM3_ESM.tif]

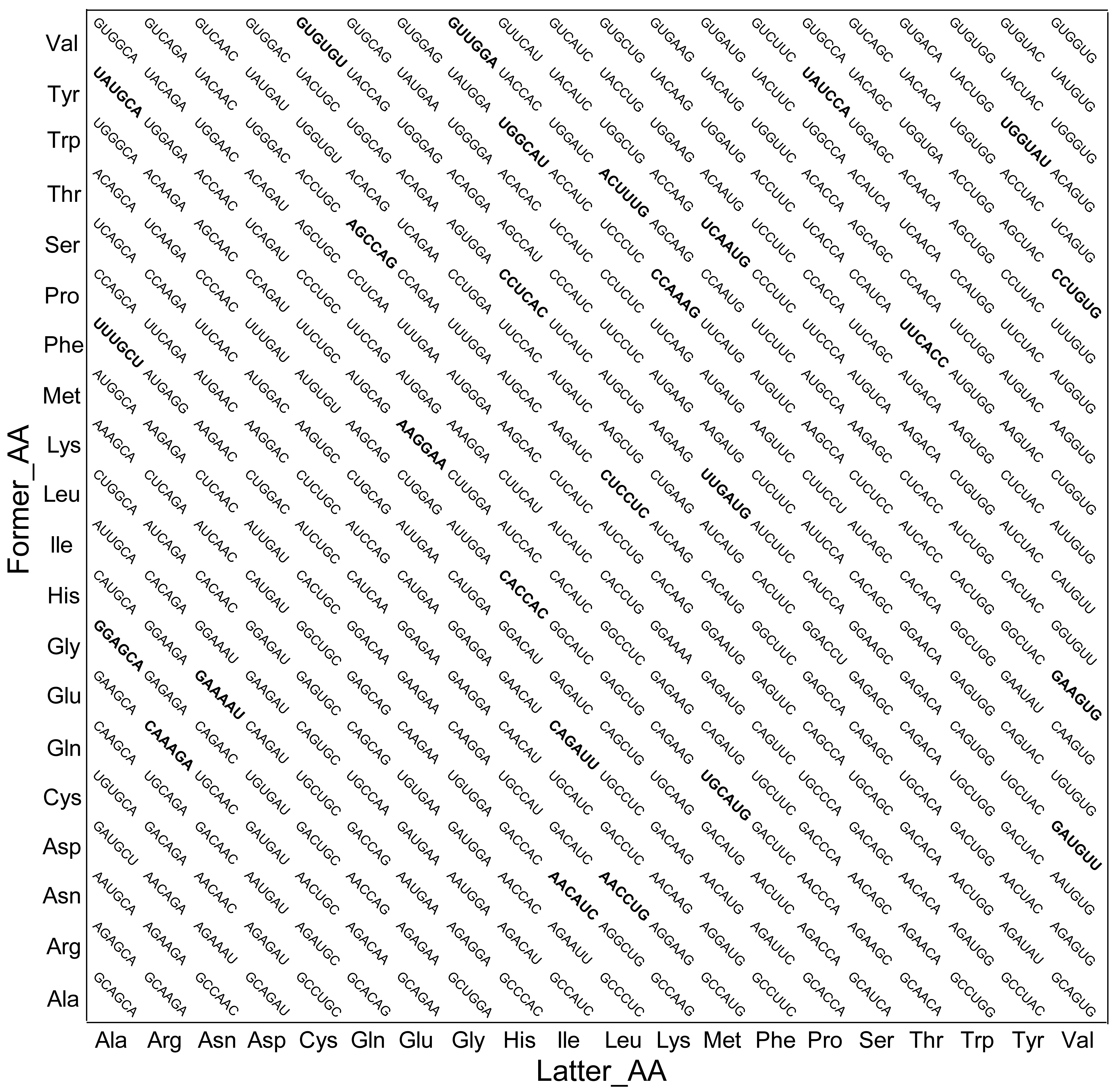

Supplement: Supplementary file 4 — Additional file 4: Fig. S4. The codon pairs of M. pulchricaudatus. [file 12864_2021_8106_MOESM4_ESM.tif]
